# Supplementary material for: Natural epialleles of Arabidopsis SUPERMAN display superwoman phenotypes
Source: Commun Biol. 2020 Dec 15;3:772. doi: 10.1038/s42003-020-01525-9 (PMC7738503; doi:10.1038/s42003-020-01525-9)
Supplement: Supplementary file 2 — Supplementary Information [file 42003_2020_1525_MOESM2_ESM.pdf]

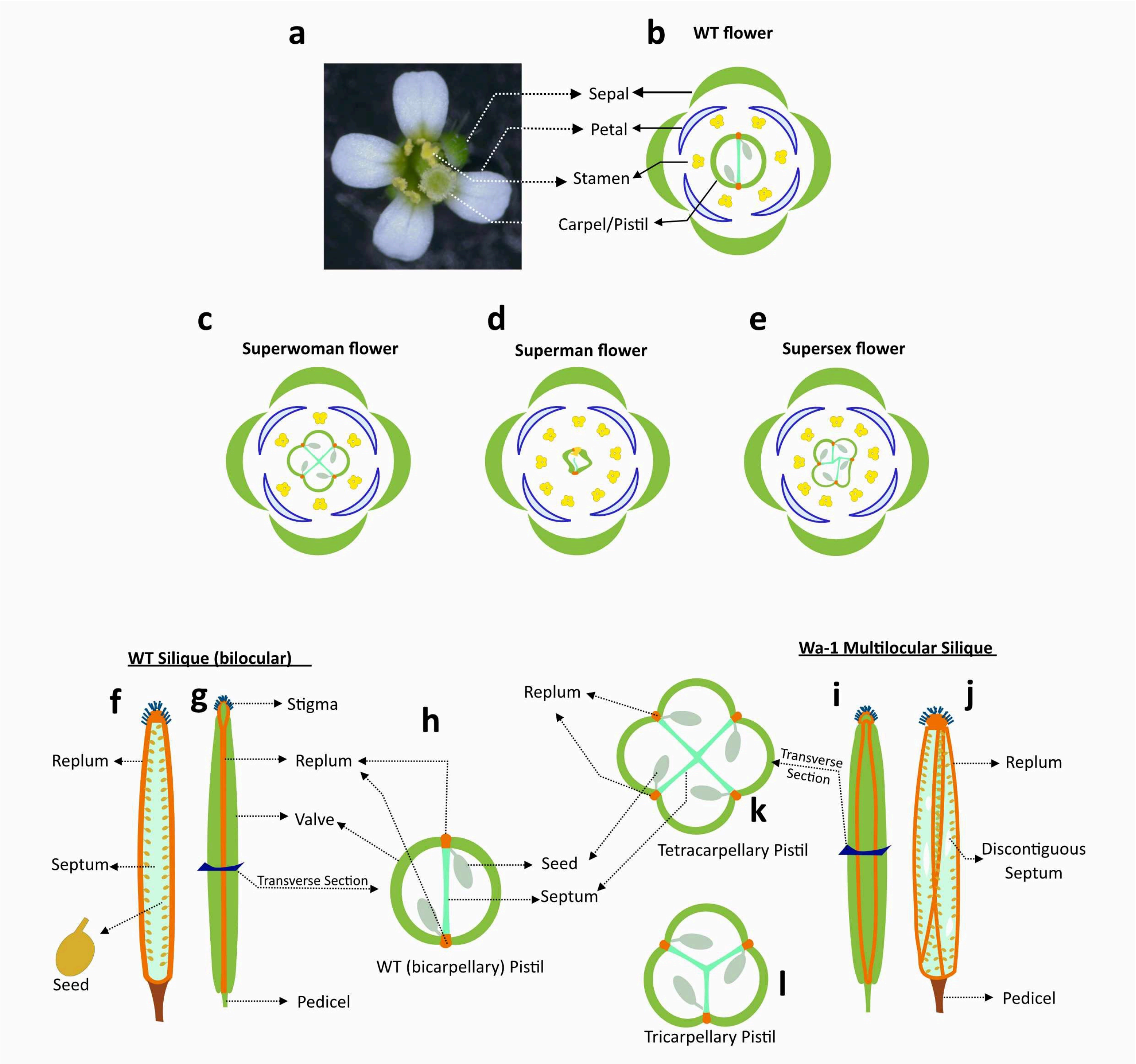


**Supplementary Fig. 1: Diagrammatic representation of wild type (WT) *A. thaliana* flower and siliques with that of *superman* mutant flowers and siliques.** **a** WT *A.thaliana* bisexual flower from Col-0 accession. **b** The floral diagram of a WT flower in *A.thaliana*. **c** The floral diagram of *superwoman* flower with first three whorls that are normal and a supernumerary carpel whorl. **d** The floral diagram of a *superman* flower with supernumerary stamen whorl at the expense of carpel whorl which is rudimentary. **e** The floral diagram of a *supersex* flower with supernumerary stamen and carpel whorl. **f-h** Cartoon representation of a WT silique arising from a bicarpellary pistil showing different parts of the silique as labelled in the diagram. **i-l** Cartoon representation of a fatty silique arising from a multicarpellary pistil showing different parts of the silique as labelled in the diagram.

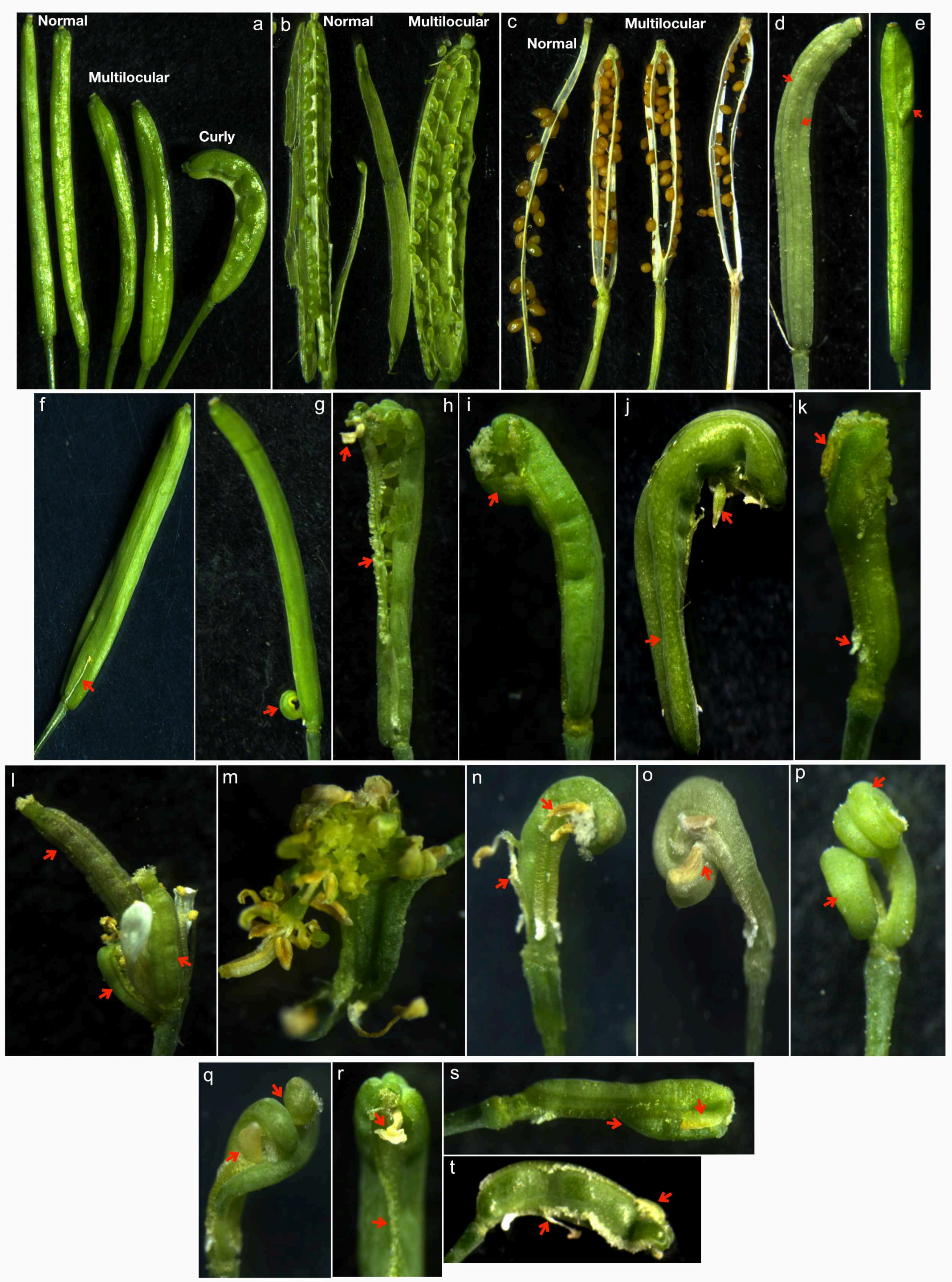


**Supplementary Fig. 2: Phenotypic characterization of *sup* epimutation in derived diploid Wa-1 plants. a** Spectrum of silique phenotypes observed in diploid(2n=2x) Wa-1 plants. **b** a normal silique originating from a bicarpellary pistil is split open to reveal two placental arrays of seeds(left image). A split view of multilocular silique arising from a tetracarpellary pistil showing four placental arrays of seeds is shown. **c** Replum-septum skeleton along with seeds captured from mature siliques with the carpel valves removed. Two rows of seeds are visible in WT Col-0 diploid, in contrast to four rows of seeds in Wa-1 multilocular siliques. **d** A multicarpellary fatty silique **e** A partial tricarpellary silique, the replum bifurcates at the proximal portion of the silique (arrow). **f** A multilocular silique with one of the anther filament remaining fused to the carpel valve(arrow). **g** A rudimentary curly silique like structure(arrow) originating from a multilocular silique. **h** A partially fused multicarpellary silique exposing ovules. The apical stigmatic tissue like structure is seen spreading all along the carpel margins(arrow). **i** A multilocular silique with apical fusion defects. **j** A curly silique with leafy outgrowth(arrow). **k** An abnormal silique with organ fusion defects. **l** A silique arising from carpel tissues(arrow) **m** A rare abnormal proliferation phenotype seen in aged/old inflorescences(2/1000 plants). **n** Organ fusion defect wherein a stamen remains fused with matured silique(arrow). **o** Abnormal curly silique. **p** Unfused tetracarpellary pistil, either bicarpellary pistil remains coiled (arrows). **q** Abnormal fusion in a tetracarpellary pistil giving a twisted finger phenotype. **r-t** A flavor of other abnormalities in the siliques of Wa-1 plants.

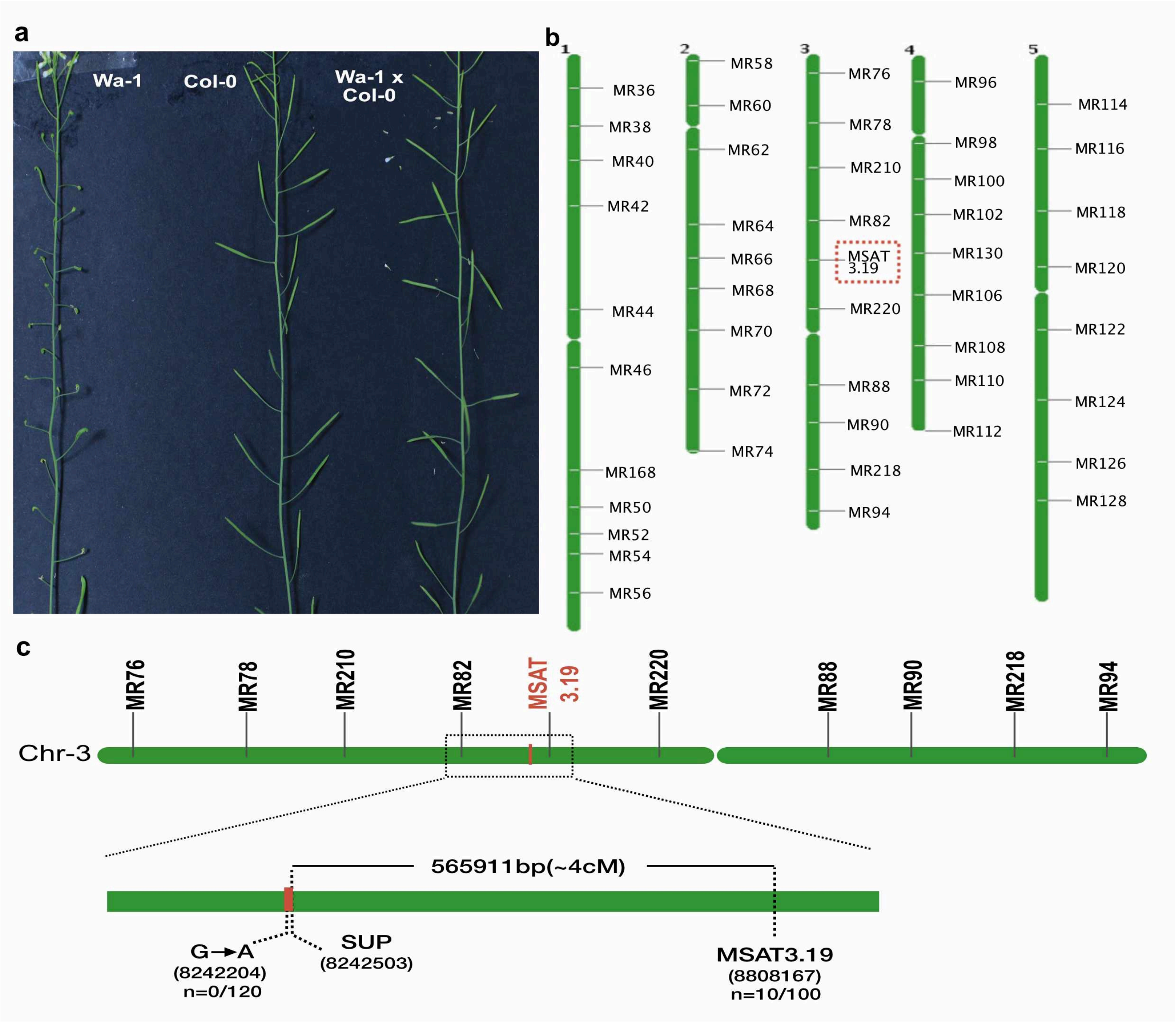


**Supplementary Fig. 3: Genetic mapping of *Wa- superwoman.*** **a** Inflorescence phenotypes of Wa-1 with curly, multilocular siliques; WT Col-0 with normal bilocular siliques and F1 hybrid from Wa-1 x Col-0 showing bilocular siliques like WT Col-0. **b** Cartoon representation of *Arabidopsis thaliana* chromosomes displaying the relative position of polymorphic markers that are used for rough mapping of *Wa-superwoman* in Wa-1 diploid plants. MSAT 3.19 marker (in the red dotted box) cosegregates with the phenotype. The details of the marker are given in the Supplementary table 4. **c** Cartoon representation of third chromosomes of *A. thaliana* showing fine mapping of *SUP* with physical distances(TAIR 10) as indicated. No of recombinants / total plants analyzed for the given marker is given in the brackets.

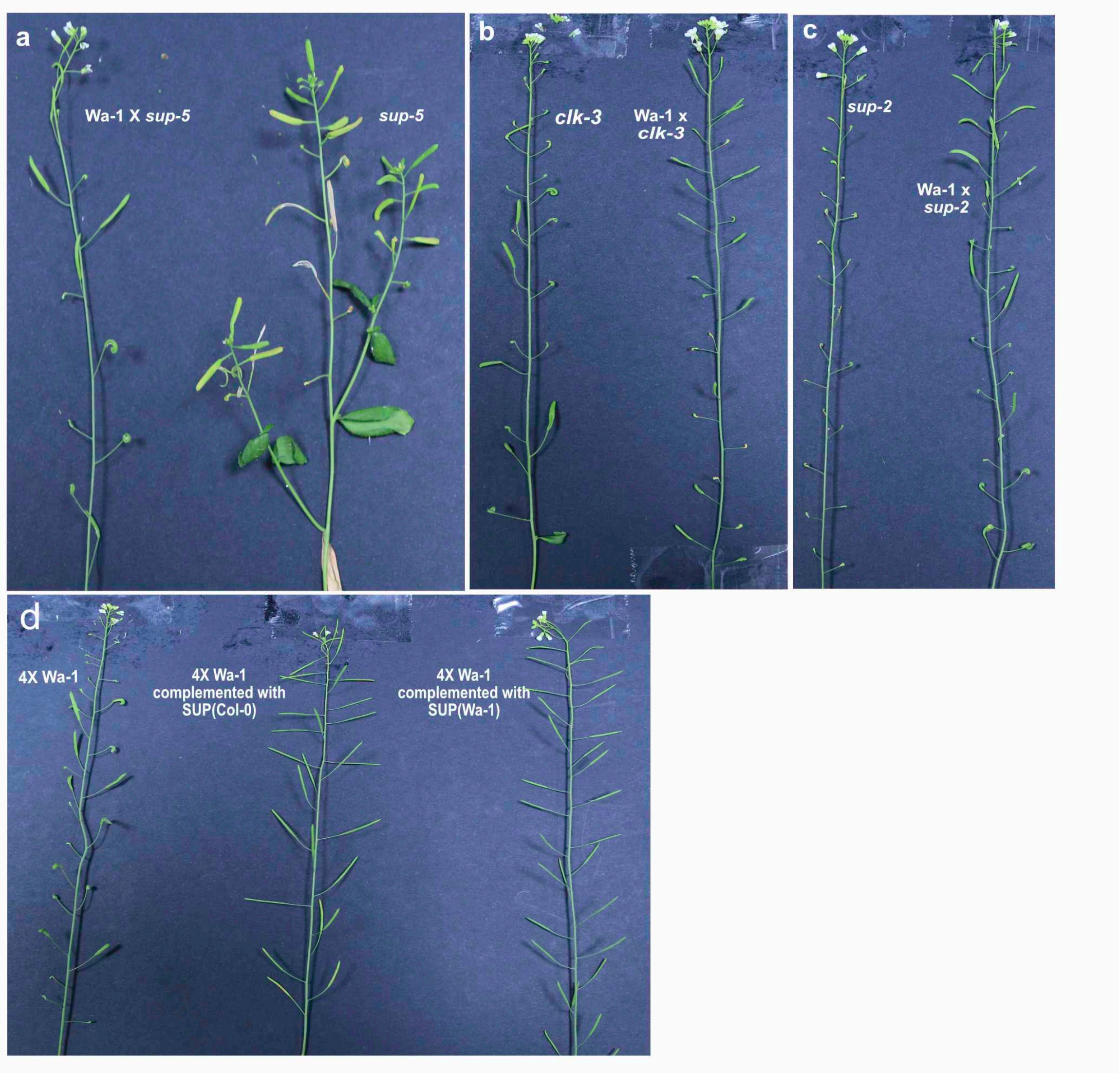


**Supplementary Fig. 4: Genetic complementation and heteroallelic phenotypes of *sup* (epi)alleles.** **a** Inflorescence phenotype of recessive *sup-5* deletion mutant along with F1 hybrid (Wa-1 x *sup-5*) showing Wa-1 *superwoman* phenotype indicating dominance of Wa-1 allele over *sup-5* allele. **b** Inflorescence phenotype of *clk-3* epimutants (*superman* and *supersex* phenotypes) along with F1 hybrid (Wa-1 x *clk-3*) showing Wa-1 *superwoman* phenotype indicating dominance of Wa-1 allele over *clk-3* **c** Inflorescence phenotype of *sup-2* showing complete *superman* phenotype along with F1 hybrid (Wa-1 x *sup-2*) showing Wa-1 *superwoman* phenotype indicating dominance of Wa-1 allele over *sup-2* genetic mutant. **d** Inflorescence phenotype of tetraploid Wa-1 with a mix of curly and multilocular siliques , alongst with inflorescences expressing ectopically inserted transgenic 6.7kb complementing genomic clone from Col-0 and Wa-1 (G to A) SNP showing restoration of WT phenotypes in both clones indicating complementation. This rules out SNP as being the cause of the floral phenotypes.

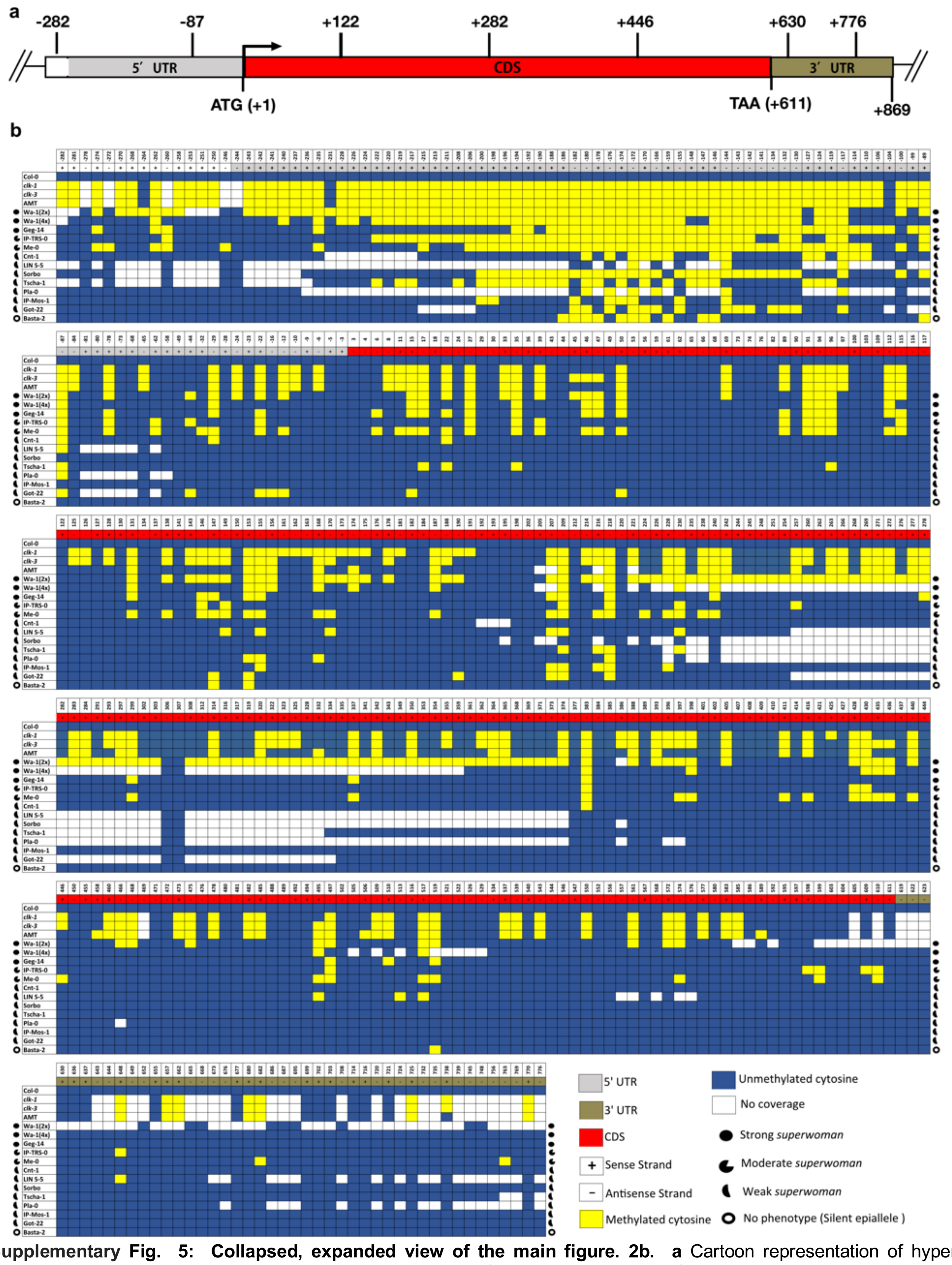


**Supplementary Fig. 5: Collapsed, expanded view of the main figure. 2b.** **a** Cartoon representation of hypermethylated SUP transcribed region. The numbers depict the relative position of DNA sequence with reference to adenine nucleotide in ATG start codon of *SUP* as +1. The numbers given in the top indicates the beginning and end of each of the 6 blocks as shown in the fig.b. **b** Methylation status of all cytosines present in the *SUP* transcribed region with its location numbered in relation to +1 ATG start codon. Individual cytosine methylation profiles for 17 genotypes are shown. Col-0 as unmethylated control, *clk-1*,*clk-3*,*AMT* induced *SUP* alleles as methylated controls (data for the figure is extracted from Fig.3 (Jacobsen & Meyerowitz, 1997)[25], 2x Wa-1 (our sequencing data), 12 accessions including 4x Wa-1 as listed in the fig. b are from the 1001 epigenomes project(Kawakatsu et al., 2016)[21]. (+) for the sense strand and (-) for the antisense strand. Methylated and unmethylated cytosines are color coded as shown in the figure legend. White spaces indicate cytosines not enough sequence coverage to call it methylated or not. The symbols at the beginning and end of each row in the blocks indicates the strength of the *sup* phenotype displayed by respective accession as shown in the legend.

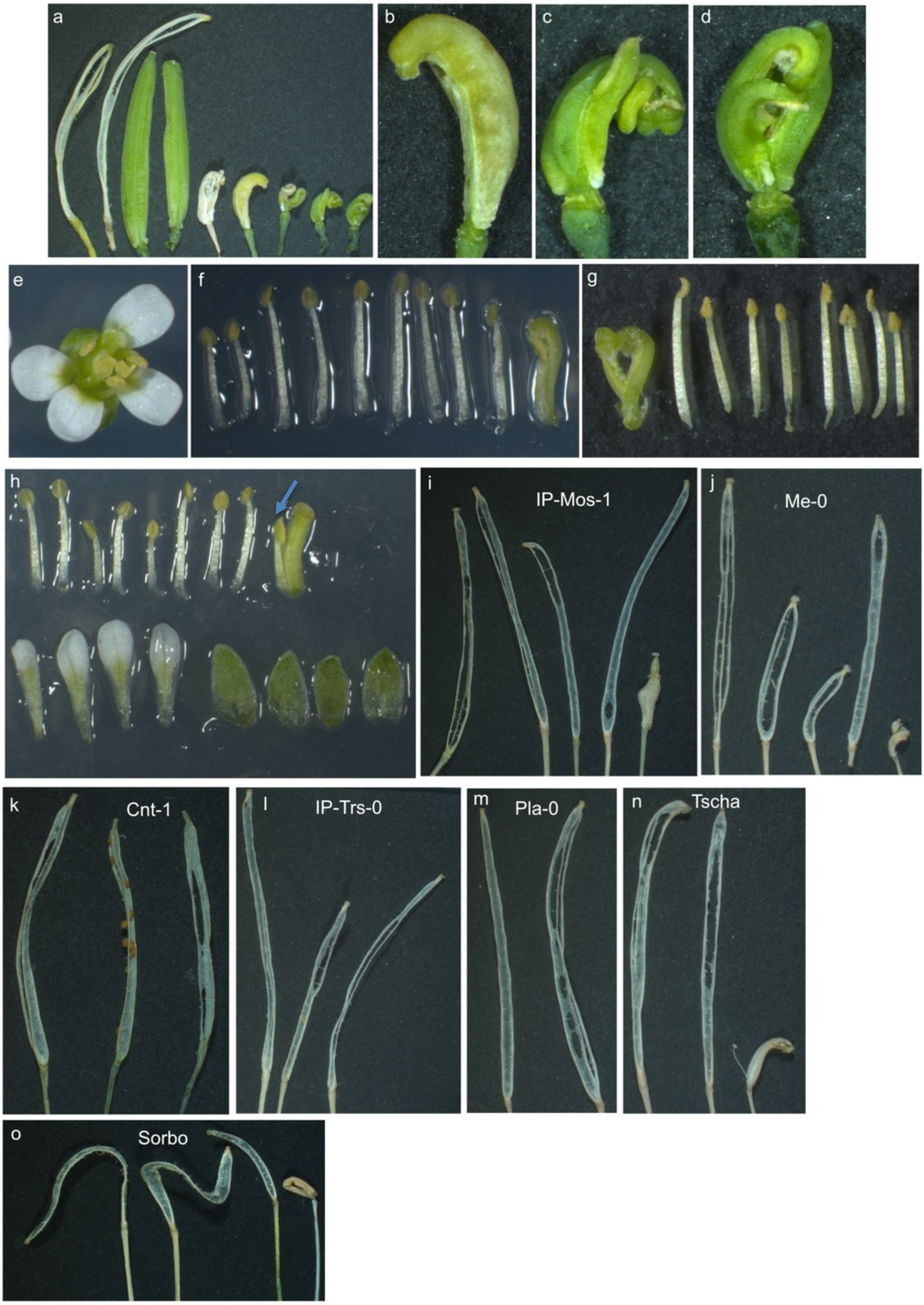


**Supplementary Fig. 6: Spectrum of phenotypes observed in natural *sup* epialleles identified from diploid *Arabidopsis* accessions. a-h** range of phenotypes observed in Geg-14 accession. **a** range of silique phenotypes showing fatty and curly siliques. **b-d** organ fusion defects in the pistil similar to tetraploid Wa-1 phenotypes. e. a *supersex* intact flower from Geg-14, which is dissected to show supernumerary nine anthers and tetracarpellary unfused silique(f). **g** dissected third and fourth whorls from a *superman* flower with 8 anthers and rudimentary carpel. **h** a *supersex* flower with all the whorls dissected. Note the staminode like anther fused with the pistil(arrow). **i-n** replum-septum skeletons remains of dehisced siliques showing superwoman phenotypes from distinct natural accessions as labelled in each panel.

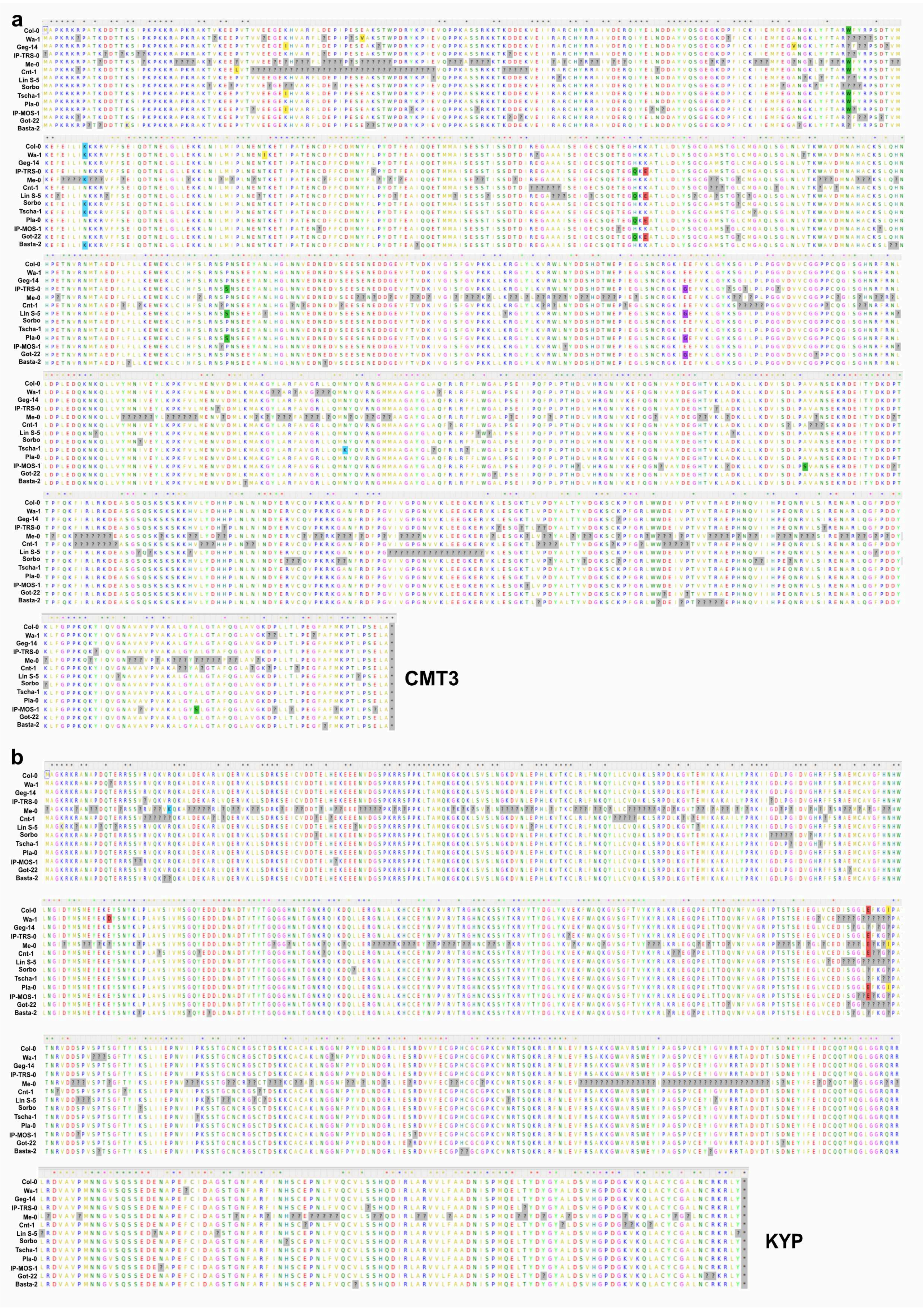


**Supplementary Fig. 7:** ClustalW alignment of the protein sequences of DNA methyl transferase CMT3(a) and histone methyl transferase,KYP(b) from the natural accessions showing *lol* phenotypes (data extracted from 1001 genomes project database). No significant shared polymorphism is common to accessions. “?” – No coverage for that amino acid.

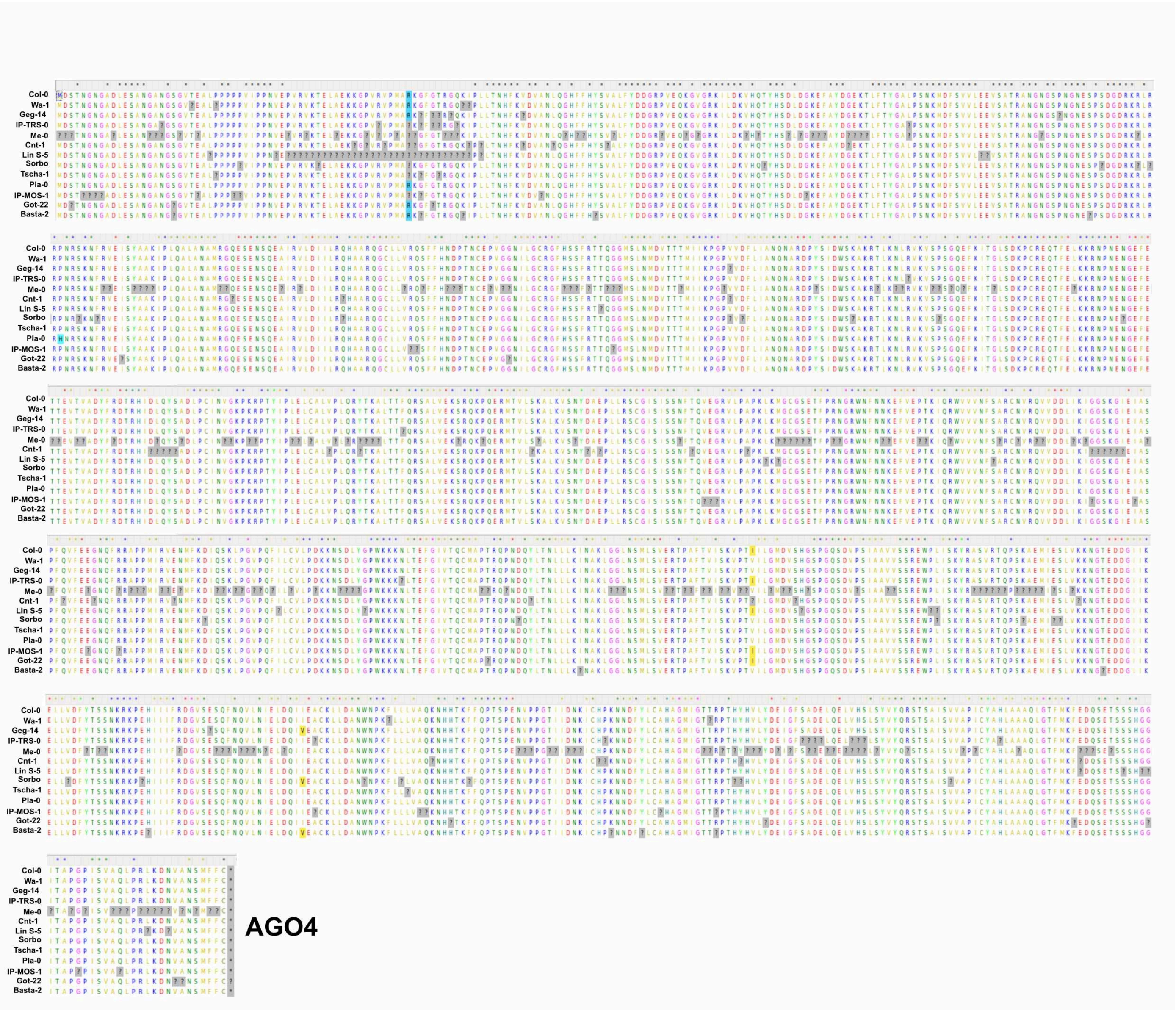


**Supplementary Fig. 8:** ClustalW alignment of AGO4 protein sequences from the natural accessions showing *lol* phenotypes (data extracted from 1001 genomes project database). No significant shared polymorphism is common to accessions. "?" – No coverage for that amino acid.

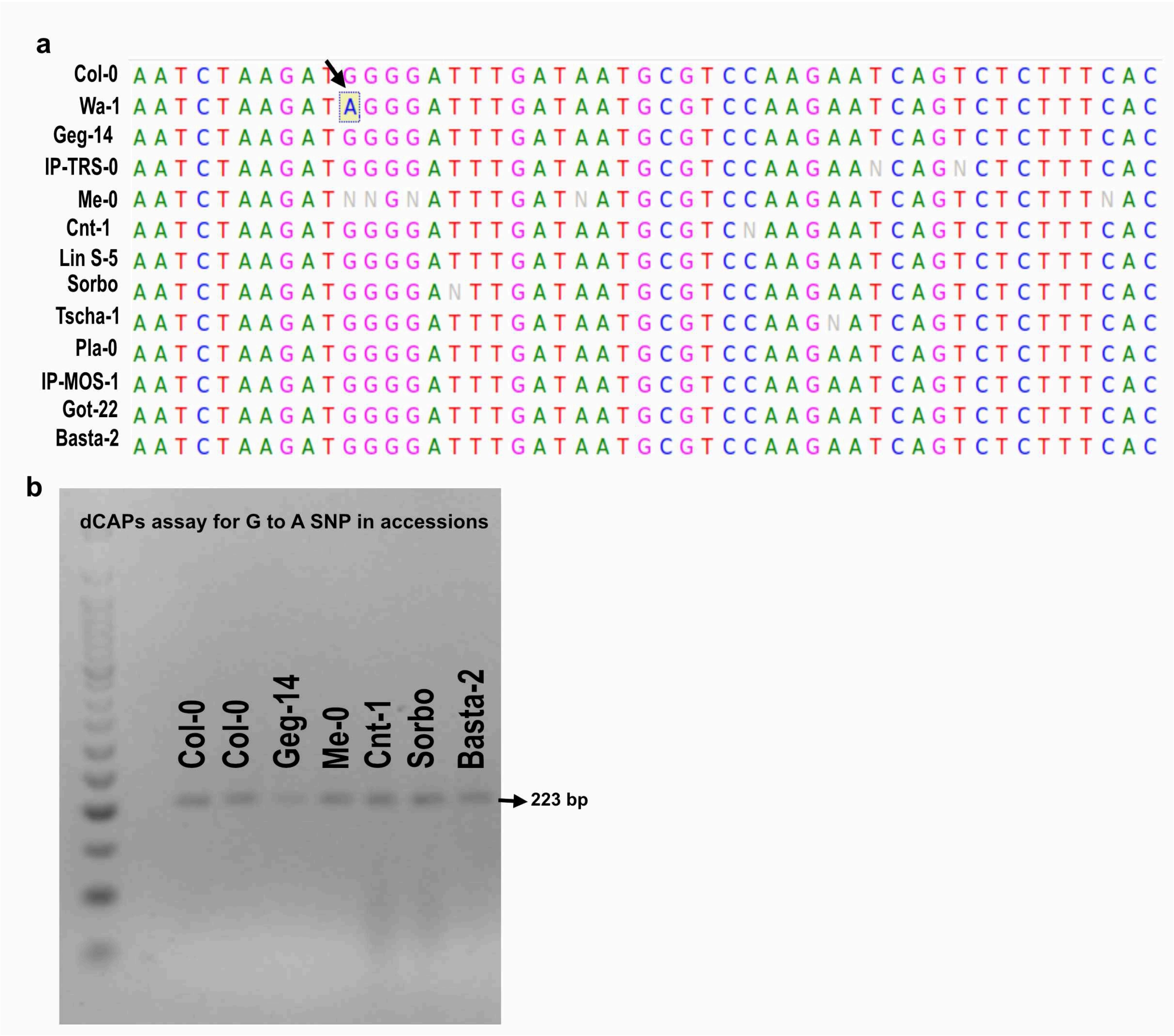


**Supplementary Fig. 9: a** DNA sequence alignment of the upstream regulatory region of *SUP* loci showing G to A SNP that is unique to Wa-1(*lol-1*) accession showing *superwoman* phenotypes, is not present in other natural accessions(*lol-2 to lol-12*). The data is extracted from 1001 genomes project database. The SNP polymorphism is indicated by the arrow. **b** Validation of the SNP by dCAPs assay. All the accessions show 223bp fragment indicating the presence of Col-0 SNP instead of Wa-1 SNP(193bp). "N" in the sequence alignment indicates insufficient coverage for that nucleotide.

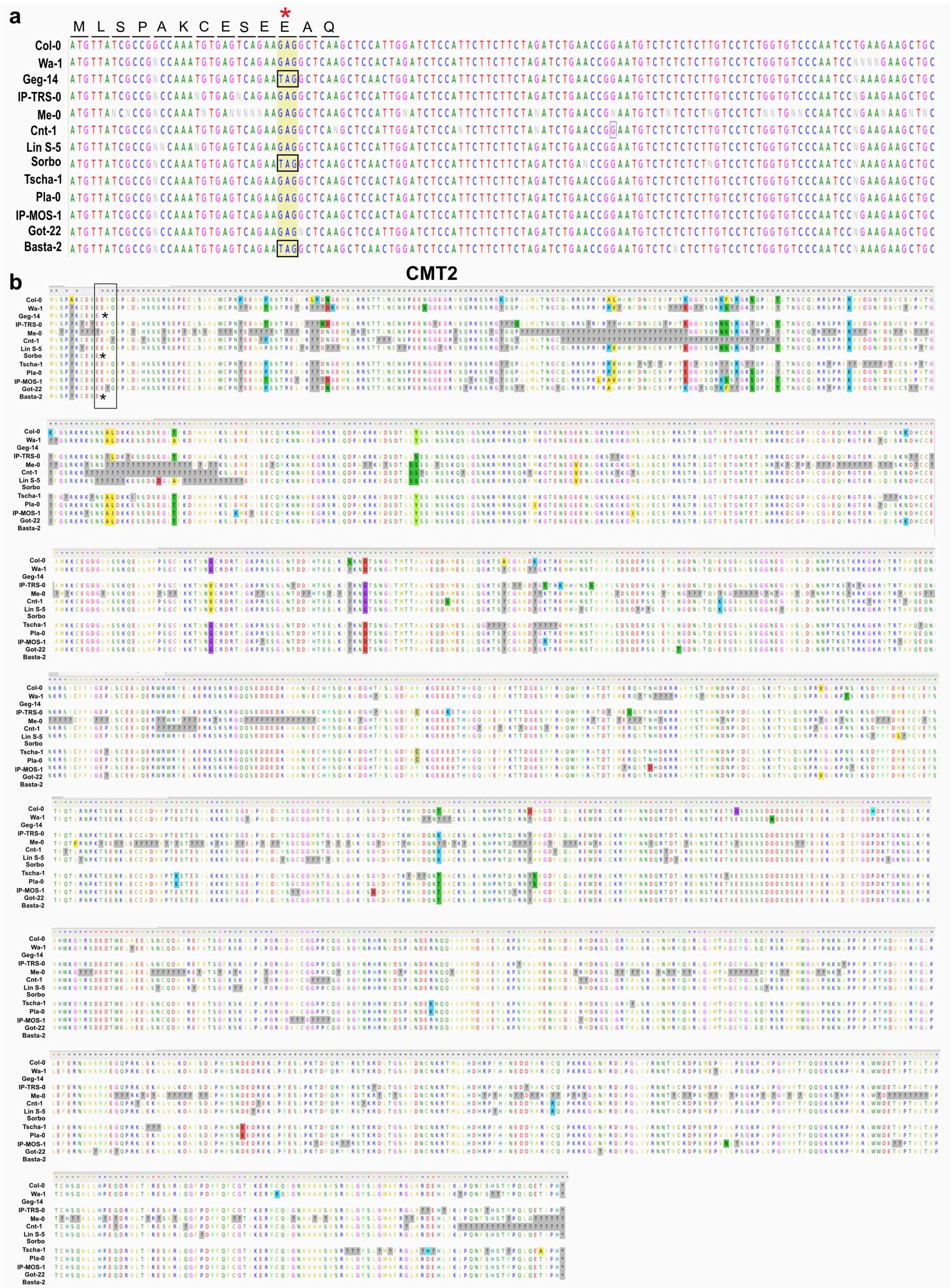


**Supplementary Fig. 10 : a** DNA sequence alignment of the starting coding region of *CMT2* gene loci showing the presence of stop codon (TAG) leading to premature truncation of *CMT2*(*cmt2*$_{STOP}$ allele) in three accessions Geg-14, Sorbo and Basta-2. **b** ClustalW alignment of CMT2 protein sequences from the natural accessions showing *lol* phenotypes No significant shared polymorphism is common to accessions. "?" – No coverage for that amino acid. The sequence data is extracted from 1001 genomes database. * indicates the position of truncation/stop codon.

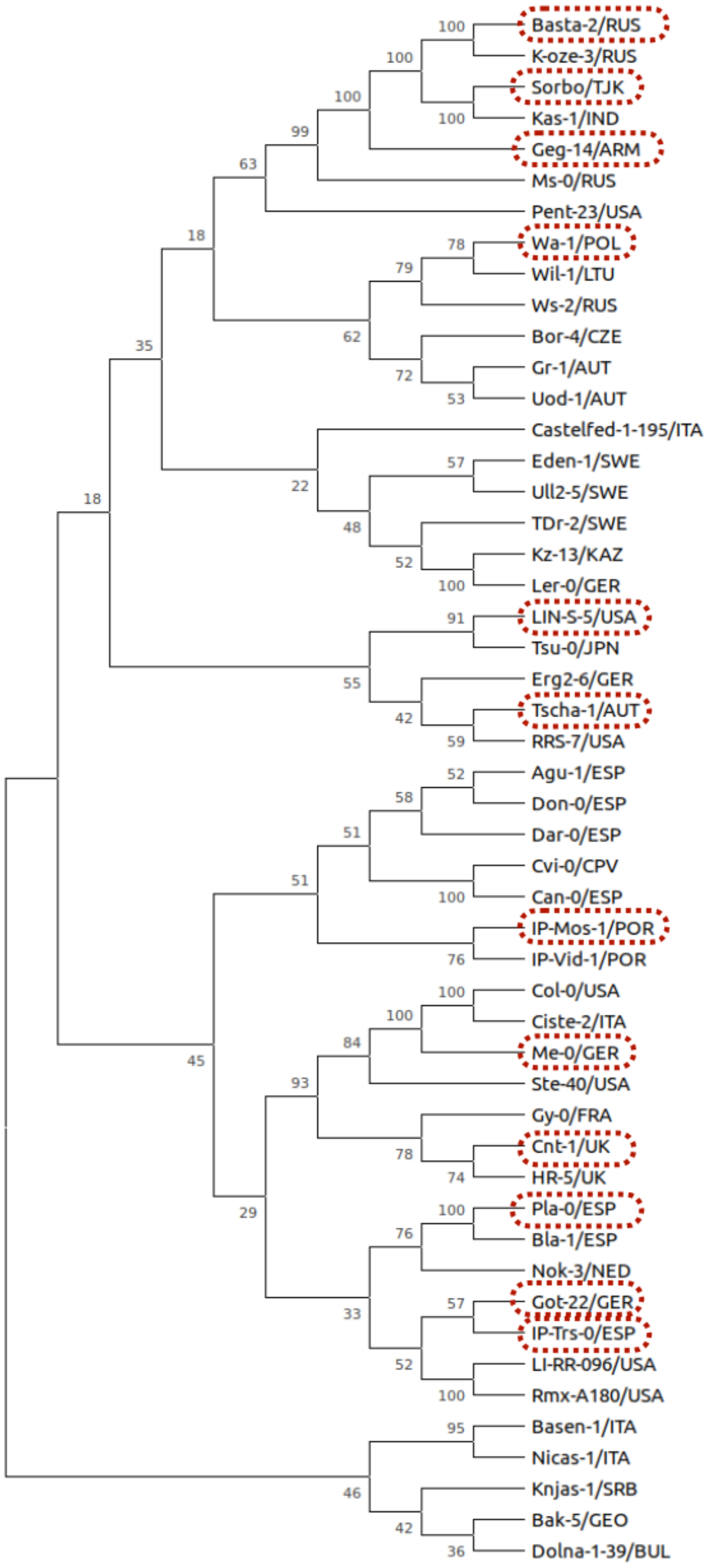


**Supplementary Fig. 11:** Phylogenetic tree comprising of 12 *sup* loci hypermethylated accessions(boxed by dotted lines) alongst other 38 geographically distinct accessions randomly chosen from each of the geographic cluster as reported in 1001 genomes project. The percentage of replicate trees in which the associated accessions clustered together in the bootstrap test(100 replicates) are shown next to the branches. The phylogenetic tree reveals that the 12 natural accessions are not genetically related ruling out a common origin.

*lol*-1(Wa-1)/ *lol*-1(Wa-1) | *lol*-1(Wa-1)/ *lol*-6(Sorbo) | *lol*-6(Sorbo)/ *lol*-6(Sorbo)

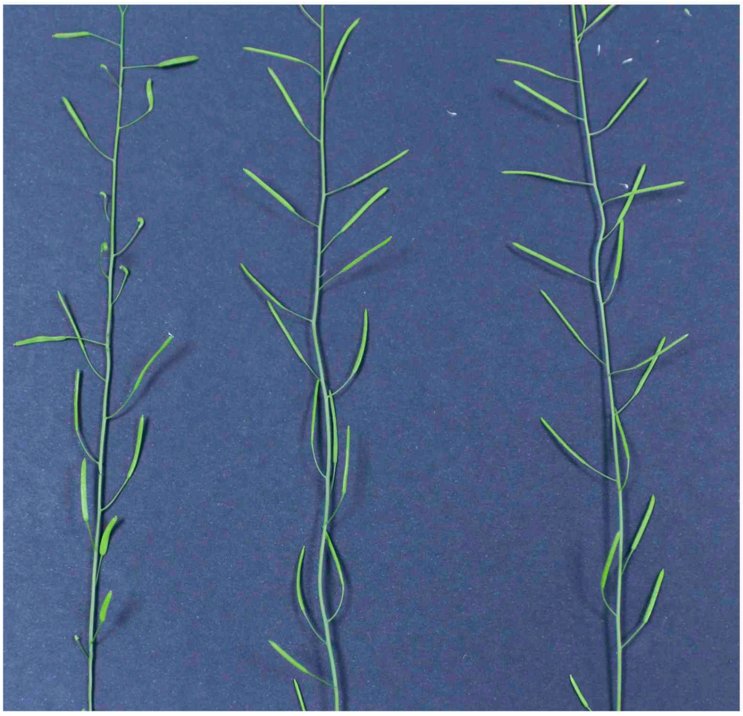

**Supplementary Fig. 12: Inflorescence phenotypes F2 segregants arising from Wa-1 (strong *lol-1* epiallele) x Sorbo( weak *lol-7* epiallele)cross.** The plants homozygous for Wa-1 show Wa-superwoman phenotype, whereas plants heterozygous for both alleles show phenotypes similar to Sorbo homozygotes indicating the dominance of weak epialleles over the strong epiallele. The genotypes are ascertained by dCAPs assay as described in materials and methods.

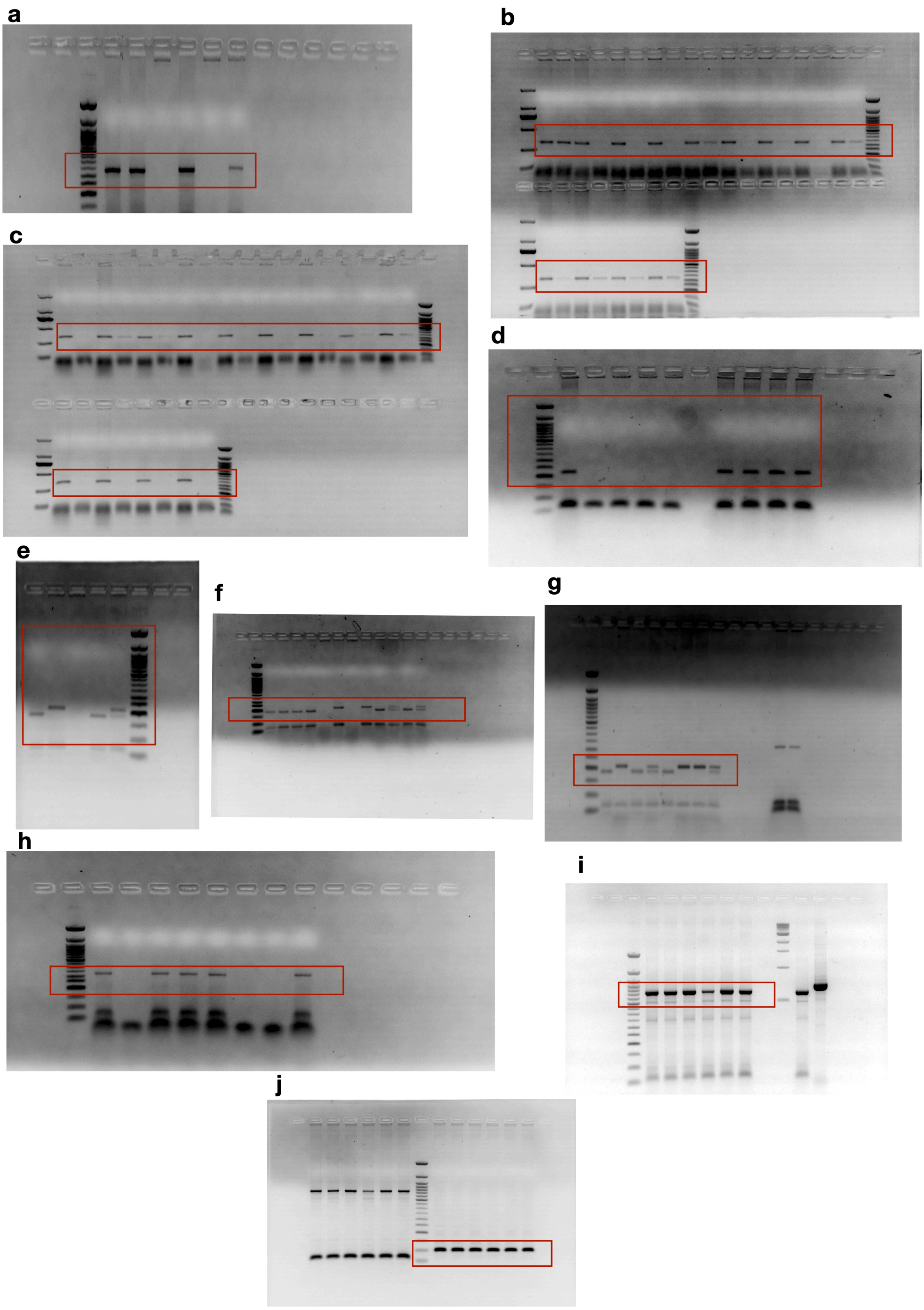


**Supplementary Fig. 13: Fig. S12**: **Uncropped gel images used in the main figures.** Corresponding lanes from the main figures are marked with a red rectangle (**a**) for Fig. 2d, **(b)** for Fig. 3m, **(c)** for Fig. 3n, **(d)** for Fig. 4c, **(e)** for Fig. 4f, **(f)** for Fig. 4g, **(g)** for Fig. 4h, **(h)** for Fig. 4i, **(i)** for Fig. 4k, **(b)** for Fig. 3m

## Supplementary Table 1: List of genetic and induced epigenetic alleles of *SUPERMAN*

| S.no | Allele | Nature of mutation | Mutagen | Ecotype/ Background | Associated mutation (if any) | Seed stock ID | Reference | Classification |
|---|---|---|---|---|---|---|---|---|
| 1 | ***sup-1/flo* 10-2** | Nonsense mutation @ Trp 22 (TGG) to a stop (TGA) | Ethyl Methanesulfonate (EMS) | L*er* | N/A | | Sakai et al.(1995); Bowman et al. (1992); | *superman* |
| 2 | ***sup-2/flo* 10-1** | Nonsense mutation @ 22 Trp(TGG) to a different stop codon(TAG) | EMS | Col-0 | N/A | CS6225 | Sakai et al.(1995); Bowman et al. (1992); | *superman* |
| 3 | ***sup-3/flo* 10-3/ *bb*4** | Missense mutation at amino acid 63 | EMS | Col-0 | N/A | CS6227 | Sakai *et al*.(1995); Bowman *et al*. (1992); | *superman* |
| 4 | ***sup-4*** | Nonsense mutation @amino acid 22 | EMS | Col-0 | N/A | similar to *sup*-1 | Sakai *et al*.(1995) | *superman* |
| 5 | ***sup-5*** | Deletion starting before the ATG, from –46 to + 568 (614 nucleotides) | EMS | L*er* | N/A | CS3882 | Gaiser *et* al.(1995); Jacobsen and Meyerowitz (1997) | *supersex* |
| 6 | ***sup-6*** | Missense mutation at amino acid 108 (Pro > His; CCT > CAT) | | Ws | N/A | | Breuil-Broyer *et al.*(2016) | *superman* |
| 7 | ***carpel*** | epiallele | EMS(co-segregated from *abi*3-4) | L*er* | N/A | | Rhode *et al.*(1999) | *superwomen* |
| 8 | ***clk*-1** | epiallele | *fwa*-1 (EMS) | L*er* | N/A | | Jacobsen and Meyerowitz (1997) | N/A |
| 9 | ***clk*-2** | epiallele | EMS | L*er* | N/A | | " | |
| 10 | ***clk*-3** | epiallele | EMS | L*er* | N/A | CS69095 | " | *supersex* |
| 11 | ***clk*-4** | epiallele | *gl*2-1 segregated | L*er* | N/A | | " | N/A |
| 12 | ***clk*-5** | epiallele | Diepoxybutane | L*er* | N/A | | " | N/A |
| 13 | ***clk*-6** | epiallele | T-DNA | L*er* | N/A | | " | N/A |
| 14 | ***clk*-7** | epiallele | *tt2* (X-ray) | L*er* | N/A | | " | N/A |
| 15 | ***fon*1-1** | epiallele | T-DNA | L*er* | *fon*1-1 was from a transformant carrying a pMON530-based construct that contains the GPA7 sense cDNA encoding a G protein alpha subunit | | Huang and Ma, 1997 | *supersex* |
| 16 | ***fon*1-2** | epiallele | T-DNA | L*er* | *fon*1-2 was from a transformant carrying a pMON530-based construct that contains the GPA7 sense cDNA encoding a G protein alpha subunit | | " | *supersex* |
| 17 | ***fon*1-3** | epiallele | T-DNA | L*er* | *fon*1-3 was from a transformant of a construct containing the maize P gene sense cDNA | | " | *supersex* |
| 18 | ***sup-eA*31** | epiallele | Ac/Ds line | L*er* | N/A | | Breuil-Broyer *et al*.(2016) | *superwoman* |

**Supplementary Table 2: Segregation analysis of Wa-1 inflorescence phenotypes on a single silique and individual plant basis**

a. Segregation analysis based on seeds collected from a single silique representing each category.

| Silique type | No. of plants analysed | No. of plants | | |
|---|---|---|---|---|
| | | Curly | Multilocular | Mix of multilocular and curly inflorescence |
| Multilocular | 45 | 21 | 6 | 17 |
| Curly | 28 | 10 | 4 | 14 |
| WT looking silique | 50 | 19 | 9 | 22 |

b. Segregation analysis based on seeds pooled on an individual plant basis representing each category.

| Inflorescence type | No. of plants analysed | No. of plants | | |
|---|---|---|---|---|
| | | Curly | Multilocular | Mix of multilocular and curly inflorescence |
| Curly | 110 | 23 | 24 | 63 |
| Multilocular | 93 | 21 | 23 | 49 |
| Mix (Multilocular+Curly) | 90 | 7 | 22 | 61 |

**Supplementary Table 3: Data on number of methylated cytosines common to natural epiallele Wa-1 and induced epiallele *clk-1* and *clk-3***

| | Total number of cytosines | Total number of cytosines Covered | Total Methylated | Number of cytosines Commonly methylated | Number of cytosines differentially methylated compared to *clk-1* | Number of cytosines differentially methylated compared to *clk-3* |
|---|---|---|---|---|---|---|
| **Wa-1(Diploid)** | 445 | 365 | 197 | N/A | N/A | N/A |
| ***clk-1*** | 445 | 383 | 205 | 152(77.2%) | 44 | N/A |
| ***clk-3*** | 445 | 383 | 210 | 158(80.2%) | N/A | 38 |
