# Supplementary material for: Natural epialleles of Arabidopsis SUPERMAN display superwoman phenotypes
Source: Commun Biol. 2020 Dec 15;3:772. doi: 10.1038/s42003-020-01525-9 (PMC7738503; doi:10.1038/s42003-020-01525-9)
Supplement: Supplementary file 3 — Description of Additional Supplementary Files [file 42003_2020_1525_MOESM3_ESM.pdf]

Description of Additional Supplementary Files

File Name: Supplementary Data 1
Description: Seed set count data for the box and whisker plot shown in Fig.1K

File Name: Supplementary Data 2
Description: Raw data for the individual methylated cytosine calls in *SUP* locus from two biological replicates, used for compiling data given in figure 2b and supplementary figure 5b.

File Name: Supplementary Data 3
Description: Percentage of cytosines methylated in different sequence contexts for the 17 genotypes shown in the bar graph of figure 2e.

File Name: Supplementary Data 4
Description: Data for the Δ^{ct} values obtained from Real time RT PCR experiment for measuring SUP mRNA levels in *lol* accessions as shown in Figure 4J

File Name: Supplementary Data 5
Description: List of Oligos used for genetic mapping of SUP locus in Wa-1 diploid accession.
